# PSYCHOSOCIAL WORKING CONDITIONS IN MID-LIFE AND COGNITIVE AND PHYSICAL IMPAIRMENT IN OLDER AGE

**DOI:** 10.1093/geroni/igac059.2274

**Published:** 2022-12-20

**Authors:** Shireen Sindi, Shadi Kiasat, Ingemar Kåreholt, Charlotta Nilsen

**Affiliations:** Karolinska Institutet, Solna, Stockholms Lan, Sweden; Karolinska Institutet, Solna, Stockholms Lan, Sweden; Jönköping University, Jönköping, Jonkopings Lan, Sweden; Jönköping University, Jönköping, Jonkopings Lan, Sweden

## Abstract

**Background:**

Psychosocial working conditions have been associated with cognitive and physical impairment among older adults. However, less is known on whether psychosocial working conditions are associated with a combination of cognitive and physical impairments. The aim of this study was to investigate the associations between midlife psychosocial working conditions and physical and cognitive impairment among older adults, and to assess whether there are sex differences in these associations.

**Methods:**

The data were derived from two Swedish nationally representative surveys (n=839) with a follow-up time of 20-24 years. Multinomial and binary logistic regressions were used to assess the associations between work stressors according to the job demand-control model, and a combination of cognitive and physical impairment.

**Results:**

Low control jobs were significantly associated with higher odds of both cognitive and physical impairment as well as a combination of cognitive and physical impairment. Passive jobs (low control, low demand) were associated with higher odds of cognitive impairment, and cognitive and physical impairment in combination. Active jobs (high control, high demand) were associated with lower odds of cognitive impairment. Sex-stratified analyses showed stronger associations among men than among women. Among men passive jobs were significantly associated with both cognitive and physical impairment. Low strain jobs were significantly associated with less physical impairment.

**Conclusions:**

These results highlight the importance of midlife psychosocial working conditions for late-life physical and cognitive impairment, and especially among men. Jobs characterised by higher control, lower strain and active jobs may promote resilience and cognitive reserve among older populations.

